# Does the Number of Dental Implants Affect Marginal Bone Loss in the Posterior Mandible?

**DOI:** 10.30476/DENTJODS.2021.90990.1542

**Published:** 2022-09

**Authors:** Reza Tabrizi, Zeynab Shaban Nejhad, Anahita Fayyazi, Hamidreza Moslemi, Shervin Shafiei

**Affiliations:** 1 Dept. of Oral and Maxillofacial Surgery, Dental School, Shahid Beheshti University of Medical Sciences, Tehran, Iran; 2 Dental Student, Dental School, Shahid Beheshti University of Medical Sciences, Tehran, Iran; 3 Prosthodontist, Dept. of Prosthodontics, Dental School, Shahid Beheshti University of Medical Sciences, Tehran, Iran; 4 Oral and Maxillofacial Surgery Resident, Shahid Beheshti University of Medical Sciences, Tehran, Iran

**Keywords:** Dental Implants, Alveolar bone loss, Prosthodontics, Mandible

## Abstract

**Statement of the Problem:**

Marginal bone loss (MBL) is an important factor in dental implant failure. The number of implants may affect MBL.

**Purpose:**

The aim of this study was to compare MBL in patients who received two or three implants for replacement of three missing teeth in the posterior mandible.

**Materials and Method:**

This prospective cohort study evaluated patients who required replacement of three missing teeth in the posterior mandible with dental implants. The patients were assigned to two groups. In the group 1, the edentulous area was restored with two implants and a pontic while three implants were placed for this purpose in the group 2. The MBL was compared between the two groups at 12 and 24 months after loading.

**Results:**

Forty-two implants were studied in group 1 and 36 implants in the group 2. The mean MBL was 0.90±0.12mm in the group 1 and 0.89±0.12mm in the group 2 at 12 months after loading. The mean MBL was 1.00±0.10mm in the group 1 and 0.98±0.10mm in the group 2 at 24 months after implant loading. The mean of MBL was not statistically different between the two groups at 12 months and 24 months (*p*> 0.05).

**Conclusion:**

It seems that the use of two or three implants for replacement of three missing teeth in the posterior mandible is not associated with an increase in MBL.

## Introduction

Rehabilitation of the posterior mandible in edentulous patients with dental implants is a commonly practiced treatment [ 1 ]. When three teeth are missing, there are two options for their replacement with dental implants: two implants with a pontic and three implants with three fixed prosthetic separate crowns. Each option has advantages and disadvantages [ 1 - 2 ]. The first option (two implants with a pontic) is cost-effective and easily applicable when the mesiodistal space is insufficient. Occlusal overloading may aggravate the marginal bone loss (MBL) [ 2 ]. In the placement of three implants, the cost of treatment increases and space management may be problematic. However, increasing the number of implants may help in better distribution of occlusal loads and decrease the MBL [ 2 ]. 

The stability of peri-implant bone is an essential parameter for the long- success of dental implants [ 3 ]. The dental implant success criteria are complex, but achieving stable osseointegration is a critical parameter in this respect [ 4 ]. MBL is a key factor in the success of dental implants. MBL≤ 2mm during the first year after functional loading is considered normal [ 4 ]. The search of the literature by the authors revealed no study comparing MBL following the aforementioned two treatment options for replacement of the lost teeth in the posterior mandible. Hence, this study was conducted to address whether the number of dental implants for the replacement of three missing teeth in the posterior mandible affect the MBL ort. 

## Materials and Method

The authors designed a prospective cohort study. The sample was derived from the population of patients presenting to the Department of Oral and Maxillofacial Surgery, Shahid Beheshti University of Medical Sciences and Khanevadeh Dental Private Clinic, Tehran, Iran for the rehabilitation of the posterior mandible with dental implants from September 30, 2015, through October 31, 2019. The Ethics Committee of Shahid Beheshti University of Medical Sciences (IR.SBMU.DRC.REC. 1398.056) approved the study. 

The patients eligible for inclusion in the study had class I skeletal relationship, three missing teeth in the posterior mandible and underwent dental implant treatment. The patients who were smokers, have been partial edentulous in the maxilla, had parafunctional activity (bruxism and clenching), had systemic diseases affecting bone metabolism, required bone augmentation, failed to show up for the follow-up, or refused study enrollment were excluded from the study.

 All implants were loaded three months after placement. TS III Osstem implants (Osstem, South Korea) were used. All prostheses were cemented type and split. One oral and maxillofacial surgeon placed all implants, and one prosthodontist fabricated the implant restorations. Digital parallel radiographs were also obtained during the study period. All radiographs were taken in the same oral and maxillofacial radiology center. Two radiology experts measured the MBL. The MBL was measured at the mesial and distal of implants by comparing the bone level on the digital parallel radiographs taken immediately after loading, and at12 and 24 months later. When the MBL was different at the mesial and distal implants, the mean MBL was calculated and reported. The bone level was measured from the alveolar crest to the fixture collar. The patients were assigned to two groups. The patients received three implants in the group 1, and two implants in the group 2. The age and gender of patients and implant diameter and length were the study variables, while the MBL was the outcome of the study. The use of two or three implants was the predictive factor of the study. An inter-examiner reliability analysis was done using the Kappa test to assess the consistency between the examiners.

## Statistical Analysis

The statistical analyses were performed using the Statistical Package for the Social Sciences for PCs, version 21 (SPSS Inc., IL, USA). The independent t-test was applied to compare the MBL, and fixture’s length and diameter between the two groups. The Chi-square test was used to compare the number of males and females in the two groups. We considered *p* Values< 0.05 as statistically significant.

## Results

Twenty-one patients with total 42 implants were studied in group 1 and 12 patients with total 36 implants in the group 2 ( Table 1). The mean age of patients was 41.24± 9.65 years in the group 1 and 39.83±11.48 years in the group 2. There was no difference in the mean age between the two groups (*p*= 0.56). A total of 11 males and 10 females were studied in group 1, and 6 males and six females were studied in the group 2. Analysis of the data did not demonstrate any difference in gender distribution between the two groups (*p*= 0.51).

**Table 1:** Descriptive of the study

Variables	Descriptive value
Age (years)	40.59±10.49
Gender	17 males ,16 females
Implant diameter (mm)	4.26±0.25
Implant Length (mm)	10.27±0.94
Groups	42 in group 1, 36 in group 2
MBL* at 12 months after loading (mm)	0.90±0.12
MBL* at 24 months after loading (mm)	1.0±0.10

*MBL: Marginal bone loss

**Figure 1:**
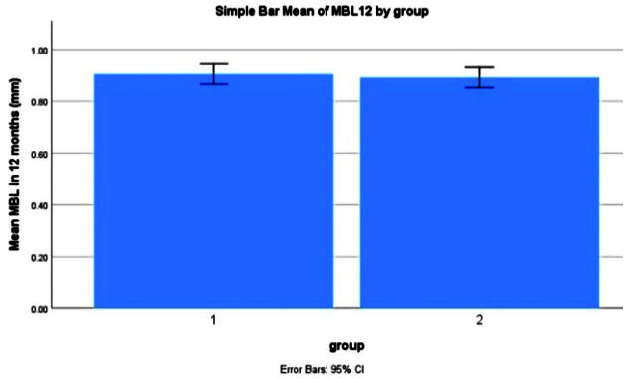
The mean of MBL in group1 and 2 in 12 months after insertion

**Figure 2:**
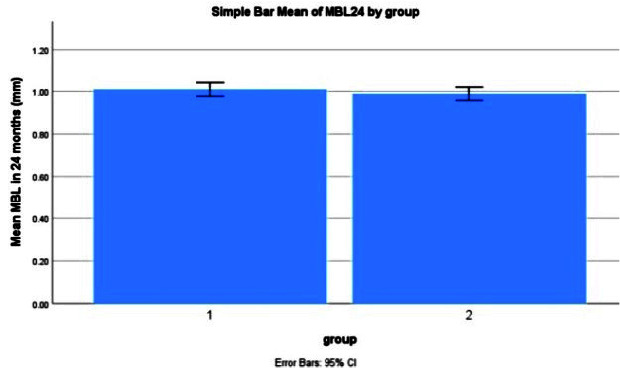
The mean of MBL in group1 and 2 in 24 months after insertion

The mean implant diameter was 4.27±0.25mm in the group 1 and 4.24±0.25mm in the group 2. There was no difference in the mean implant diameter between the two groups (*p*= 0.51). The mean implant length was 10.40±0.89mm in the group 1 and 10.11±0.98mm in the group 2. Statistical analysis did not indicate any difference in the mean implant length between the two groups (*p*= 0.17, Table 2). The mean MBL was 0.90±0.12mm in the group 1 and 0.89±0.12mm in the group 2 at 12 months after loading (Figure 1). There was no significant difference in the mean MBL between the two groups at 12 months after loading (*p*=0.63). The mean MBL was 1.00±0.10mm in the group 1 and 0.98±0.10mm in the group 2 at 24 months after implant loading (Figure 2). Analysis of the data did not demonstrate any difference in the mean MBL between the two groups at 24 months after loading (*p*= 0.35; Table 3). The inter-examiner reliability was kappa=0.52 (*p*=.0.008) at 95% CI, which indicated a moderate agreement between the observers.

**Table 2:** Comparison of variables between the two groups

Variables	Group 1	Group 2	*p* Value
Age (years)	41.24±9.65	39.83±11.48	*p*= 0.56*
Gender	11 males, 10 females	6 males, 6 females	*p*= 0.51**
Implant diameter (mm)	4.27±0.25	4.24±0.25	*p*= 0.51*
Implant length(mm)	10.40±0.89	10.11±0.98	*p*= 0.17*

*Independent t-test **Chi-square test

**Table 3:** Comparison of MBL in 12 and 24 months after implant loading

Outcomes	Group 1	Group 2	Independent t-test
MBL at 12 months after loading	0.90±0.12	0.89±0.12	*p*= 0.63
MBL at 24 months after loading	1.0±0.10	0.98±0.10	*p*= 0.35

## Discussion

MBL is among the important factors in the long-term success of dental implants [ 5 ]. Several local and systemic factors are responsible for MBL [ 6 ]. The number of implants is considered as an influential factor for the reduction of MBL [ 2 ]. In this study, MBL was assessed in two treatment options for replacement of three missing teeth in the posterior mandible including two implants with a pontic and three implants. Assessment of the M-BL in the two treatment options can help clinicians to develop an acceptable treatment plan for similar situations.

This study indicated that MBL was not different in the use of two or three implants for the replacement of three missing teeth in the posterior mandible. Tabrizi et al. [ 2 ] studied the MBL around short implants in the posterior mandible. Their findings contradicted our results, reporting that the MBL decreased with an increase in the number of short implants. The possible reason for the difference in the results of the two studies can be the crown to implant ratio [ 7 ]. Another study reported that the crown to implant ratio did not play a role in the increase of MBL [ 8 ]. Early MBL is due to the remodeling process of bone, which is not related to infection. Early MBL occurs one year after dental implant placement [ 9 ]. In addition, infection-related MBL occurs in peri-implantitis [ 10 ]. Surgical factors (insufficient bone volume, implant malpositioning, bone overheating during implant site drilling, and extreme cortical compression) and prosthetic factors (the type of implant-abutment connection, implant-abutment microgap, residual cement reaction, and early loading) can all affect the MBL [ 11 - 13 ].

Several studies support overloading as a factor responsible for increased MBL [ 11 - 13 ]. Occlusal overload is defined as the application of loads greater than the withstanding capability of the implant or prosthetic components or the surrounding tissues [ 11 - 13 ]. Minor occlusal overload does not cause MBL [ 14 ]. In the placement of two implants with pontic, occlusal overloading does not occur if sufficient bone volume is available, and implants are placed in a correct position. An excessive dynamic load results in crater-like bone defects lateral to the osseointegrated fixtures [ 14 ]. It is unclear whether occlusal overload might be a cause of MBL or not [ 15 ]. Moreover, higher remodeling activity of the peri-implant bone occurs around implants under high loading forces [ 15 ]. It should be noted that bone quality, implant diameter and implant surface characteristics affect MBL around implants; it is reported that a poor bone quality, a smaller diameter of implants, and a smooth surface adversely affect MBL [ 16 ].

In this study, we considered implant diameter and length as the variables of the study. As the implant diameter was not different between the two groups, it cannot be responsible for any possible difference in MBL. It has been reported that narrow fixtures may be associated with higher MBL [ 17 ]. Surgeons may have more confidence in the treatment outcome when a higher number of implants are placed. However, financial issues and anatomical limitations may prevent the placement of the maximum number of implants. 

## Conclusion

It seems that the use of two or three implants for replacement of three missing teeth in the posterior mandible is not associated with an increase in MBL.AcknowledgementAll procedures performed in studies involving human participants were in accordance with the ethical standards of the institutional and/or national research committee and with the 1964 Helsinki declaration and its later amendments or comparable ethical standards. Informed consent was obtained from all individual participants included in the study.

## Conflict of Interest

The authors declare that they have no conflicts of interests.
